# Failures in Protein Clearance Partly Underlie Late Onset Neurodegenerative Diseases and Link Pathology to Genetic Risk

**DOI:** 10.3389/fnins.2019.01304

**Published:** 2019-12-05

**Authors:** John Hardy

**Affiliations:** ^1^Department of Neurodegenerative Disease and Reta Lila Weston Laboratories, UCL Queen Square Institute of Neurology, London, United Kingdom; ^2^UK Dementia Research Institute, UCL Queen Square Institute of Neurology, London, United Kingdom

**Keywords:** Alzheimer’s disease, Parkinson’s disease, progressive supranuclear palsy, APP, MAPT, SNCA

## Abstract

As we identify the loci involved in late onset neurodegenerative disease, we are finding that the majority of them are involved in damage response processes. In this short review, I propose that it is partly a failure in these damage response processes which underlie late onset disease and that the resultant pathology is a marker of the type of damage response which has failed: microglial clearance of damaged neuronal membranes in Alzheimer’s disease (AD), ubiquitin proteasome clearance in the tauopathies, and lysosomal clearance in Parkinson’s disease (PD). In this review, I outline this relationship. This article is not intended as a comprehensive review of the cell biology of any of these disorders but rather a summary of the evidence that the genetics and pathology of these disorders appear to point, in each case, to the removal of misfolded proteins as a critical process in disease pathogenesis.

## Introduction

Three of the most common neurodegenerative diseases are Alzheimer’s disease (AD), characterized by Aβ plaques and tau tangles, the primary tauopathies, [frontotemporal dementia with tau pathology (FTD), progressive supranuclear palsy (PSP), and corticobasal degeneration (CBD)] characterized by tangle pathology and Parkinson’s disease(s) (PD), usually characterized by synuclein Lewy bodies. In all three disease categories, the autosomal dominant diseases can be caused by gene duplications (Singleton et al., 2003; Rovelet-Lecrux et al., 2006, 2010) or by mutations which make protein deposition more likely either by changing the cleavage profile of Aβ so that a less soluble form is released (Scheuner et al., 1996) or by altering the splicing pattern of the MAPT gene so that more of the four repeat isoform of tau is produced (Hutton et al., 1998). These findings show that one way of getting disease is through overloading neurons with slightly too much of these proteins. These mutations, while illustrative and useful in terms of modeling the diseases, are rare, and the majority of the cases of disease do not have these simple etiologies and, with these other cases, the relationship between the pathology and the genetic causes and predispositions has been much less clear. The finding that a slight overload of particular proteins can cause disease, suggests that problems in the clearance and metabolism of these proteins may also increase disease risk. While the clearance and metabolism of each of these proteins is likely to be complex, with the increasing number of identified genetic loci, it seems as if many of them are involved in protein clearance. Thus, the genetic evidence supports the view that protein and damage clearance is critical to disease etiology and further suggests that the proteins which are deposited are those whose clearance mechanisms are failing (Ciryam et al., 2016). This suggestion supports the view that potentiation of these clearance mechanisms may constitute a rational approach for drug discovery efforts (Boland et al., 2018).

## Alzheimer’s Disease

Missense mutations in APP or in PSEN1 and PSEN2 cause early onset autosomal dominant disease. These mutations either push more APP down the β-secretase pathway resulting in more Aβ production or alter the position of the final γ-secretase cleavage such that a greater proportion of longer, less soluble Aβ are the final product of APP processing. These mutations all make the process of Aβ deposition more likely (Hardy and Selkoe, 2002).

Genetic analysis of late onset AD has now identified over 40 loci. The majority of them are myeloid genes, probably microglial, or involved in lipid metabolism (Jones et al., 2010) or both. Many of these risk loci are part of a module of gene expression driven by the transcription factor SPI-1 which gene, PU.1, is itself a risk locus for disease (Huang et al., 2017). Most of these loci are also part of a gene expression module that responds to Aβ deposition in APP transgenic mice (Salih et al., 2018). APP metabolism occurs in the membrane compartment and many of the loci such as TREM2 are membrane lipid receptors or lipid transporters such as ABCA7 (Quazi and Molday, 2013; Wang et al., 2015). In general, the variants that are found associated with disease are loss of function variants. These data thus suggest the genes are involved in the clearance of APP/Aβ induced membrane damage and that some increased risk is associated with failings in this clearance.

There is thus a symmetry to the variants involved in AD: they either lead to overproduction of Aβ processed from APP, or some of them are in the microglial genes involved in clearing Aβ and damaged membranes.

## The Primary Tauopathies

Besides the MAPT gene duplication referred to above (Rovelet-Lecrux et al., 2010), the other mutations in MAPT which cause FTD either increase the proportion of four repeat tau by altering the splicing of exon 10 of the gene (Hutton et al., 1998), or by reducing the binding of tau to microtubules and thus increasing the concentration of free tau protein (Hong et al., 1998). Genetic analysis of the sporadic tauopathies shows that the MAPT gene itself is a major determinant of risk (Baker et al., 1999; Höglinger et al., 2011). The sporadic tauopathies (PSP and CBD) are much rarer than AD, the genetic analyses… genome wide association studies and exome sequencing… have been much less extensive and the number of loci which have been identified is correspondingly less. This means our conclusions on this group of diseases have to be more circumspect. Among the four or five loci which have been identified are STX6 and EIF2AK3, which are involved in the unfolded protein response and ubiquitin proteasome system, respectively (Höglinger et al., 2011), and TRIM11 which is also involved in the ubiquitin proteasome system (Jabbari et al., 2018). In transgenic mouse studies, potentiation of the ubiquitin proteasome system has been shown to clear tangles (Myeku et al., 2016): thus it seems parsimonious to suspect that the genetic variability in components of proteostasis systems reflect a reduced ability to degrade unfolded tau protein. It seems likely that, by analogy with the role of Aβ in AD, that mutations which increase the concentration of the free tau are one case of disease, and some of the predisposition to sporadic disease is encoded by loci involved in the clearance of this protein. Other degradative pathways for tau protein have been suggested (Cotman et al., 2005; Garg et al., 2011; Chesser et al., 2013) and while these are currently not supported by the genetic data, this may reflect the paucity of these latter data.

## Parkinson’s Disease(S)

In the majority of cases of PD, synuclein is deposited as Lewy bodies (Spillantini et al., 1997). Gene duplications of the SNCA gene are one rare cause of disease (Singleton et al., 2003) and normal genetic variability at the SNCA locus contributes to disease risk with those with higher expression at increased risk (Farrer et al., 2001). Many genes identified through analysis of mendelian PD are lysosomal (Karabiyik et al., 2017), as are many of the risk loci identified by exome sequencing and genome wide association studies (Robak et al., 2017). Again, in general, the risk loci are loss of function variants. Synuclein is degraded though the lysosome and increased expression of synuclein inhibits lysosomal flux (Mazzulli et al., 2011), so here too, there is a relationship between the pathologic protein, synuclein, and the genetics of the disease. Over production can cause disease and deficiencies in its degradative pathways can also lead to disease.

With PD, however, another set of genes, including Parkin and PINK1 are in the mitophagy pathway (McWilliams and Muqit, 2017). This is the process by which damaged mitochondria are removed through the lysosome system. These mitochondrial fragments are another lysosome substrate. In the pure, Parkin form of this disease, the pathology is does not involve Lewy bodies (Doherty et al., 2103), but in other forms of the mitophagy disease, there is Lewy body pathology suggesting that the mitophagy and synuclein degradative pathways converge on the lysosome.

## The Implications of the Failure to Degrade Hypothesis

Most work on neurodegenerative diseases has focused on the deposited proteins and whether of themselves, the deposits have particular toxic properties. The analysis presented above suggests an alternative view, consistent with the suggestion of others (Freer et al., 2016), that a rather more fundamental explanation could be that it is the age related degradative failure which largely underpins the common forms of disease and that the deposited proteins are the most obvious manifestation of that damage response and clearance failure. In each case the deposited protein seems as if it can behave in a templating manner and spread through the CNS. One could imagine that this pathology spread contributes to the spreading of the failure to metabolize. This view of disease is more concordant with the epidemiology of the diseases and the fact that they are age related. A homoeostatic system which is gradually failing as it accrues damage fails critically in one component: those with a weak microglial response, fail with amyloid deposition: those with a weak ubiquitin proteasome response, fail with tau pathology and those with an inadequate lysosome clearance process fail with Lewy body pathology. What does this suggestion imply for therapy? Substrate reduction… reducing the pressure on these clearance systems makes sense as, of course, do approaches to increase the efficiency of the clearance mechanisms. Prevention, through the reduction of the age related damage should also be a goal and inadvertent success in this area may underlie the clear reduction in dementia incidence over the last 30 years as smoking and other cardiac risks have been reduced (Chibnik et al., 2017). Anti-amyloid therapies have not so far convincingly worked for AD and while it is clear that the reasons for many of these failures may relate in part to the details of their execution (e.g., Karran and Hardy, 2014), clearly we should constantly question their underlying assumptions. I would now suggest we consider their pathogeneses as a damage response failures. This is not to say that the deposited proteins are not important but to suggest we should think of them, in part, as markers of more general failures.

## Author Contributions

The author confirms being the sole contributor of this work and has approved it for publication.

## Conflict of Interest

The author declares that the research was conducted in the absence of any commercial or financial relationships that could be construed as a potential conflict of interest.
